# The Hospitals of Madrid: a "Busman's Holiday"
1Being part of a Presidential Address to the Bath and Bristol Branch of the British Medical Association, delivered on June 22nd, 1927.


**Published:** 1927

**Authors:** E. W. Hey Groves

**Affiliations:** Senior Surgeon, Bristol General Hospital; Professor of Surgery in the University of Bristol


### The Bristol Medico-Chirurgical Journal

*"Scire est nescire, nisi id me
Scire alius sciret."*

WINTER, 1927.

### THE HOSPITALS OF MADRID: A "BUSMAN'S HOLIDAY."[1]

BY

E. W. Hey Groves, M.S., F.R.C.S.,
*Senior Surgeon, Bristol General Hospital;*
*Professor of Surgery in the University of Bristol.*

It would require courage and optimism on the part of a newly-elected President to claim your interest and attention in this full summer tide on a purely professional subject.

I count it as one of the happiest things which ever came to me in my professional career that I was elected a member of the Chirurgical Club. This Society of twenty-five surgeons, representative of all the extra-metropolitan teaching schools of Great Britain, has now visited the majority of the great centres of surgical activity in Europe, and its members have been able to

[1] Being part of a Presidential Address to the Bath and Bristol Branch of the British Medical Association, delivered on June 22nd, 1927.

see foreign methods of operating, teaching and research, and to discuss the merits of such methods with one another.

This year the Club had decided to visit the surgical clinics of Madrid during the week following Easter, and it seemed to me that a motor run through France and Spain offered great attractions which might well be added to the more strictly professional advantages of the trip. Probably most of you have motored in France, and therefore I will speak very briefly of the tour through that country, confining myself chiefly to the Spanish portion of the journey.

From Bristol it is most convenient to travel via Southampton, and we went from that port to St. Malo and returned through Havre. In each case the cross-Channel journey is by night. We arrived at St. Malo at 10 a.m. on April 9th, and were on the road within the hour, after going through the various Customs regulations and filling up with "essence."

It is a curious fact, to which I think all motorists will assent, that the change in the rule of the road presents so little difficulty. One seems to fall in with the new habit as a matter of course, though it is possible that in emergencies, when one is guided by instinct rather than reason, one might be liable to mistakes.

The shortest overland route from the north of France to the Spanish border is from St. Malo to Irun, through Nantes, Saintes and Bayonne, in stages of 122, 130 and 191 miles. Lunch and tea were generally taken by the roadside, making each day into a long picnic. The direct road to the south of France is perhaps a little monotonous, but in the spring of the year it is adorned with all the glories of the trees and fields in their springtime garb. The French roads have certainly been much improved during the last few years, and many of the main roads are now tarred. In going south from Bordeaux it is wise patiently to trace out the by-roads advised by the touring agents, so as to avoid about thirty miles of bad *pavé*. In any case the last stage of the journey through France is through the flat country of "Les Landes," a huge pine forest, every tree of which has many cups attached to its trunk to collect the resin.

It was on the morning of April 12th that we crossed into Spain, after making a short detour from Bayonne to Biarritz, and thence through the very attractive town of St. Jean de Luz, which nestles at the foot of the Pyrenees, and has attracted quite a large colony of English.

The Spanish Customs presented no difficulties at Irun, thanks to the papers provided for us by the A.A. We had to buy a local passport which lasted one month, for the sum of 10 pesetas.

The first town in Spain reached by this route is San Sebastian, and it certainly offers a happy introduction to the English visitor. It is a well-built town with esplanade and harbour on the Bay of Biscay, and every kind of modern shop ; it also has a fisherman's quarter, where a great trade in sardines is carried on. It is a favourite holiday resort of the King of Spain, who has a modern residence on the sea front. On a recent occasion of political crisis it is said that His Majesty motored from San Sebastian to Madrid, a distance of 317 miles, in one night, and by so doing was able to avert a *coup d'état*.

The first part of the road from San Sebastian to Vittoria is along the sea coast as far as Deva, and it is probably one of the best and most beautiful motoring roads of Europe. It has a good tarred surface with well-engineered curves and tunnels cut in the rock at points of danger, so as to provide a two-way road.

The rocky coast of the ocean is on one side of the road with beautiful verdant hills on the other. At Deva the road goes inland, and our reluctance to say good-bye to the sea was modified by the fact that the road goes up a fine gorge which carries down a rushing torrent and rises by an easy gradient to the Arlaban Pass.

The journey to Madrid was broken at Vittoria and Burgos, and on the homeward trip at Valladolid and Santander. At all of these places the hotels were excellent, with bathrooms generally attached to the bedrooms, willing service and good food. Difficulties in hotel accommodation would arise if through any breakdown it were impossible to reach a large town. We were most fortunate in the weather throughout the entire trip; except for one mountain mist which we went through on the return journey, we had cloudless sunshine the whole time, and heat which was genial without being oppressive. The country between Vittoria, Burgos and Madrid was grand and impressive, and its beauty was much increased by the bright sunshine.

The mountains of the Guadarrama Range, which at that time were snow covered, always gave an impressive background to the picture. The road, with a good surface, wound between many rock-bound gorges and climbed fairly steep passes. Often it was possible to see a distance of twenty miles in every direction, over an undulating country, the red and purple soil being variegated by the green crops. The greatest drawback to the country is the absence of trees, a condition which is said to have been deliberately brought about in order to exterminate the birds which feed upon the crops.

Villages in this northern Spain are quaint and picturesque. They usually lie just off the main road, and consist of a collection of mud-walled houses grouped round a church, all the buildings seeming to be huddled together for warmth and protection. The country-side gives the impression of great solitude and loneliness. For miles around the eye looks in vain for any house, man or beast. This impression is probably quite illusive; villages are hidden away behind hills and in hollows, and peasants who are quite unobtrusive to the passing motor-car soon make themselves known to the unwary loiterer who sits by the wayside for a meal.

Burgos is the nearest town of any size north of Madrid, which is 150 miles distant. After traversing the many miles of undulating plain, in which human habitation is hardly perceptible, it almost produces a feeling of shock when one suddenly enters the confines of the capital, with the multitudinous trams, buses and motor-cars, its efficient police and highly-regulated traffic.

Madrid is on a great plain over 2,000 feet above sea level, at about the mid point of Spain. It was only made the capital of Spain in the sixteenth century by that Philip the Second who ruled over the Spanish Empire when it was at the zenith of its power, and whose only great failure was his attack on England with the Invincible Armada.

It is therefore lacking in the ancient buildings and historic associations which are found so richly in all the other great cities of Spain. On the other hand, it has all the charm of a modern city, with wide streets, beautiful gardens, modern shops and the great picture galleries, which contain many of the most famous pictures in the world.

The week spent in Madrid itself was the true "busman's holiday." We arrived on April 15th, and the rest of the Club came by rail on the evening of

Easter Sunday, April 17th, rather hot and tired after thirty-six hours in the train. Each day thereafter was spent in a varying round of hospital inspection, sight-seeing, and the enjoyment of great and generous hospitality. Meal-times are usually late—3 p.m. for lunch and 9.30 for dinner—so that the day's activities seldom end before midnight.

For three mornings from 9 to 1 we watched operations in the older hospitals, the Princessa, the University and Madrid School Hospitals. Professor Cardinal and his assistants, Dr. Blanc and Dr. Slocker, did general surgery with a great predominance of gastric work, gastro-enterostomy and the various types of Polya's gastrectomy being performed several times on each of our visits. Dr. Cifuentes did genito-urinary operations, and Professor Recasens, the Dean of the Medical Faculty, gynecological. It would be wearisome if I were to relate details of all these operations, and I will only comment on those special features in the general surgical arrangements which were of particular interest.

It was noticeable that women nurses played a very small part in the surgical work. The Nursing Sisters, who attend all the large hospitals, act chiefly as sick attendants, cleaning the wards and feeding the patients. They take very little part in the operating theatre, except that of fetching and carrying. This state of affairs does not arise from the idea that the surgeons do not recognise the value of trained nurses, but rather from the difficulty of getting enough women of the right class to take up nursing. We actually went over a most up-to-date institute, named after Queen Maria-Christina, which is devoted to the training of nurses, and all the arrangements for lectures and demonstrations and those of the private hospital of which the Institute is a part were most ideal. The nurses trained in this institute at present only work for private patients and nursing homes.

The medical institution which impressed us most of all was that devoted to the treatment and re-education of injured workmen. It is situated in a country estate on the outskirts of Madrid, and is supported by the State and the insurance companies. The urgency of this need for provision against industrial accidents is emphasised by illustrated propaganda, the text of which reads: "Every minute and five seconds there occurs an accident; every hour, nine minutes and fifty seconds a workman is thrown out of employment; every seven hours, six minutes and fifty seconds a workman is killed; every half second a peseta is lost by an industrial accident."

In the institute every treatment is given to the injured workmen, beginning with surgical reconstructive operation, including some of the finest kineplastic amputations we had ever seen. Artificial limbs are fitted when necessary. When physical restoration is complete, the patient is tested by elaborate psychological reactions, to gauge his or her mentality. Re-education is then undertaken in one of the workshops until the injured person is able to return to the old work, or to earn his living by a new occupation more suitable to his altered condition. The excellence of this ideal and the success with which it is being carried into reality filled us with admiration, and I know that I am voicing the opinion of Sir Robert Jones, who was one of our party, when I say that it is well worth a visit from all interested in the surgical or industrial problems of reconstructive surgery and professional re-education.

Madrid has the same hospital problem to solve that confronts other big cities, viz. that of the modernisation of old buildings. It has been decided that it would be unwise to try and improve the existing city hospitals, because this would involve great expense, while leaving the hospitals in unsuitable city sites and multiplying general hospitals, each of which would have to be provided with special departments. Instead of this a fine site has been secured on the outskirts of Madrid on high ground which looks right out towards the Guadarrama Mountains. On this there is to be built a University city, which will include a single great University Hospital. The first portion of this new hospital is now nearing completion, and we walked through its wards and corridors with envy in our hearts.

Our Club was greatly honoured by a Royal Command to a reception at the Palace, when His Majesty, King Alfonso XIII., the Queen, who is also an English Princess, and the Queen-Mother, spoke to us each personally, and seemed interested in our impressions of Spain and Spanish surgery.

Of course, we all found an opportunity of witnessing the far-famed bull-fight, which, however objectionable may be some of its features, has an intense and vivid interest. The immense stadium, holding 20,000 spectators, packed to its utmost limit; the gay cavalcade of horsemen and matadors and other fighters who enter and circle round in the brilliant sunshine; the bull-ring empty, save for a few men with scarlet clothes; the door thrown open for the entry of the rampant bull, who soon spies his enemies and makes things lively for them; the appearance of a few sorry, broken-down horses, mounted by picadors carrying short spears; the horrid spectacle of these horses being done to death by the bull, who at the same time receives the spear thrusts in his neck; the thrilling encounter between the feverishly charging bull and the matador, who leaps aside at the moment when the beast almost has him impaled, and plants his two darts in the bull's neck; the last act, when the espador, armed only with a cloak and a sword, plunges the latter into the bull's heart; and the dragging out of the dead bull by four mules—all these incidents, which have been described by many writers, occupy about half an hour, and the drama is repeated six times during a single performance.

The return motor trip was through Valladolid and Santander, then along the glorious coast road back to San Sebastian, and home through the centre of France and the port of Havre. Certainly for those who want a motor holiday with every delight of scenery and variety nothing could be more enjoyable.

This, then, is an example of what I mean by a "busman's holiday" for a surgeon. Such holidays have been spent in the United States, Canada, and in most European countries. A "busman's holiday" is not a mere joy ride—it also consists in watching how another fellow gets the best out of his bus, how he deals with his traffic problems and brings his fares safely to their destination. In regard to hospital organisation and equipment, good nursing and perfect team work, we have perhaps been most favourably impressed by what we have seen in Canada, Denmark and Switzerland. There we have found hospitals which are just as perfect as modern knowledge knows how to make them—adequate financial support both in regard to capital expenditure and maintenance; all classes of the community, from the richest to the poorest, equally well provided for; medical science placed on the same footing as other branches of learning, so that young men are trained and selected, men of experience are placed in charge, and the complexities of a great science are served by the best possible team of men working together, both for the discovery of new truth and the best application of knowledge for the cure and prevention of disease.

